# “It is what I tell her that she will do”: a mixed methods study of married men’s knowledge and attitude towards supporting their wives’ cervical cancer screening in rural South-East Nigeria

**DOI:** 10.11604/pamj.2020.36.156.21157

**Published:** 2020-07-06

**Authors:** Ijeoma Nkem Okedo-Alex, Chigozie Jesse Uneke, Henry Chukwuemeka Uro-Chukwu, Ifeyinwa Chizoba Akamike, Onyedikachi Echefu Chukwu

**Affiliations:** 1 African Institute for Health Policy and Health Systems, Ebonyi State University, Abakaliki, Ebonyi State, Nigeria,; 2 Department of Community Medicine, Federal Teaching Hospital Abakaliki, Ebonyi State, Nigeria

**Keywords:** Attitude, cervical cancer screening, knowledge, male involvement, married men, Nigeria

## Abstract

**Introduction:**

cervical cancer is a leading cause of death among Nigerian women. Women often require spousal support before attending cervical cancer screening services. This study assessed married men´s knowledge and attitude towards male involvement in cervical cancer screening of their wives.

**Methods:**

a cross-sectional study using a mixed methods approach was conducted among 245 married men in Izzi, Local Government Area of Ebonyi State, South-East Nigeria. Quantitative data collected using structured, interviewer-administered questionnaires and qualitative data from focus group discussions were triangulated. Data analysis was done using IBM SPSS version 20. Qualitative findings were analysed using thematic analysis.

**Results:**

the mean knowledge of cervical cancer was 2.06±0.55. Only 2.9% of the respondents had adequate knowledge of risk factors for cervical cancer. Up to 89.8% were willing to approve screening for their spouses. Majority (76.3%) considered screening important in cervical cancer prevention, while 91.4% were willing to pay for the screening test. Most of them exhibited patriarchal tendencies and insisted that their wives must obtain their consent before screening as depicted by the statement “It is what I tell her that she will do”. Previous spousal screening was a predictor of good knowledge (OR = 10.94, 95% CI = 2.44-48.93; P=0.002).

**Conclusion:**

married men in this study had poor knowledge of cervical cancer. However, they were willing to support cervical cancer screening conditional on their pre-information and consent. Awareness creation activities on cervical cancer screening should incorporate active engagement of husbands in order to promote screening uptake by their wives.

## Introduction

Nigeria is one of the top five countries with the highest mortality of cervical cancer by absolute numbers globally. Cervical cancer ranks as the 2nd most frequent cause of cancer deaths among women in Nigeria and also the third leading cause of cancer deaths among women aged between 15 and 44 years of age [1, 2]. Even though the Human Papillomavirus Virus (HPV) vaccines are effective in preventing HPV infection and the development of cervical cancer, the vaccine is frequently not accessible or cost-effective for women in less developed countries. Thus cervical cancer prevention in these countries rely mostly on secondary prevention via screening [3]. Men could participate in reproductive health either as clients of Reproductive Health (RH) services themselves; as supportive partners for women´s reproductive health and/or as agents of change within the community [4]. Thus men can play important roles in the prevention and treatment of cervical cancer by encouraging HPV vaccination, screening and treatment by their partners/wives, sisters and mothers. This is because they are often referred to as the gatekeepers of access to health services for the women in their lives [5].

In developing countries with low uptake of cervical cancer screening, women have identified lack of spousal support as a barrier to screening [6]. This is especially so in rural patriarchal African societies where a significant proportion of the women depend on their husbands for material and financial sustenance [7]. Poor spousal support towards screening for cervical cancer could stem from low knowledge regarding the scourge and risk of the disease. However, only few studies have assessed the knowledge and attitude of men towards cervical screening by their wives and partners. These studies have found that men have limited knowledge and misconceptions regarding cervical cancer and its detection by screening. Most of these studies also showed that men were willing to support screening by their wives and partners [6, 8-10]. To the best of our knowledge, no such study has been conducted among Nigerian men. Findings from this study would be useful in designing programs to promote uptake of cervical cancer screening by married women. The objective of this study was to assess the knowledge and attitude towards cervical cancer screening among married men in a rural community in South-East Nigeria.

## Methods

**Study setting:** the study was conducted in Nwezenyi, a rural community located in Izzi Local Government Area of Ebonyi State, South-Eastern Nigeria. The people of Nwezenyi are mostly Ibos, the dominant tribe of South-East geo-political zone of Nigeria. The predominant occupations of the inhabitants of the Nwezenyi community include farming, trading and making crafts. The Izzi local dialect is the common language in the community. Nwezenyi is host to a comprehensive practicing centre annex (primary health centre) of the Federal Teaching Hospital Abakaliki (FETHA). Nwezenyi community has a men´s association comprised mainly of married men who were 245 in number. This association has existed for many generations and consists of men belonging to different age categories culturally known as age grades.

**Study design:** this was an observational cross-sectional study that employed a mixed methods approach. The quantitative data was collected using a structured interviewer-administered questionnaire. Focus Group Discussions (FGDs) were used to collect the qualitative data. Findings from the quantitative and qualitative methods were triangulated.

**Study population:** the study population were husbands of women who were at least 18 years old, resided in Nwezenyi community and belonged to the men´s association in the community. Men whose wives had been diagnosed with/ treated for cervical cancer and those who did not give informed consent were excluded. None of the married men met the criteria for exclusion and thus were all eligible for the study. A total population survey of all the 245 married men who belonged to the Nwezenyi men´s association was conducted. Participants were recruited in a non-randomised manner as the total population of the married men in the men's association were surveyed. The participants in the FGD were selected with the aid of the executive leaders of the men´s association. The FGD participants were selected on the basis of being vocal, willing to participate and express their views freely.

**Data collection methods and tools:** the researcher and five trained research assistants carried out the data collection. The research assistants were resident doctors from the Department of Community medicine, FETHA. They were trained on the specific objectives of the study, content of the different study tools, how to administer the questionnaire and facilitate FGDs. The training was held at the seminar room of the department of Community Medicine FETHA and lasted for 3 hours. Following literature review, a structured interviewer-administered questionnaire was developed and adapted from a previous study [11]. The questionnaires and results from these studies were used to develop the questionnaire employed in this study. The questionnaire included sections on socio-demographic characteristics, knowledge of cervical cancer and screening and attitude towards screening and male involvement in cervical cancer screening. Knowledge of cervical cancer and cervical cancer screening was measured on a 5-point Likert scale using 4 questions. These questions assessed self-rated knowledge on risk factors, clinical features and use of screening tests for early detection of cervical cancer. Three of the questions assessed adequacy of knowledge and the answer choices were “grossly inadequate=1”, “inadequate=2”, “fairly adequate=3, “adequate=4”, and “very adequate=5”. One of the questions assessed knowledge using agreement with a statement on a Likert scale. The answer choices to the question were “strongly agree =5”, “agree = 4”, “undecided=3”, “disagree = 2” and “strongly disagree = 1”

Attitude towards male involvement in cervical cancer screening was measured on using a total of 7 questions on a 5-point Likert scale. Four of these questions assessed willingness to support cervical cancer screening, two assessed the importance of screening while one question assessed the perception of spousal risk for cervical cancer. The answer choices of the Likert scaled questions on willingness were “strongly willingly =5”, “willing = 4”, “undecided=3”, “unwilling = 2” and “strongly unwilling = 1”. The Likert scale questions on importance were ordered as follows: “grossly unimportant=1”, “unimportant =2”, “neutral=3”, “important=4” and “very important=5”. Each questionnaire took approximately 7 minutes to fill. All the questionnaires administered were retrieved giving a response rate of 100%. All retrieved questionnaires were analysed.

There were two focus group discussions with the participants aged between 30-65 years. Each FGD had 8 participants and lasted for about 50 minutes. The discussions were conducted in Ibo language (Izzi dialect) which was the primary language of the participants. The FGD took place in the house of the chairman of the association as was unanimously agreed upon by the participants before time. Before the onset of the FGD, the moderator welcomed the participants, obtained their written informed consent to participate and assured them of the confidentiality of their responses. Ground rules guiding the discussion were also set as suggested by the participants themselves. Some of the rules involved restriction of chorus answers, mobile phone use, side talks and respect for each other´s opinion. The FGD was held in a location (house of one of the leaders) as agreed upon by all the respondents. To promote anonymity, each participant was assigned a number with which they were referred to throughout the course of the discussion.

A topic guide was used to facilitate the discussions. The FGD Guide was used to explore knowledge and attitudes of the respondents towards cervical cancer screening and male involvement in cervical cancer screening and the factors affecting their involvement in cervical cancer screening. The questions covered by the guide include: What do you know about cervical cancer? How can it be transmitted? Can cervical cancer be prevented? Do you think cervical cancer screening can prevent cervical cancer? Do you think that husbands should be actively involved in promoting cervical screening by their spouses? What are the ways men can be involved in cervical screening by their spouses? What other ways men can help prevent cervical cancer? The research assistants for the FGD were the moderator, electronic record handler and a note taker. The primary author was the moderator of the FGD. An electronic audio recorder was also used to record the discussions and field notes made by the note taker. Verbatim transcription from the electronic recorder was done within 24 hours after each session and compared with the notes from the note-taker.

**Data management/analysis:** to assess the knowledge and attitude towards cervical screening among the men, the Mean Neutral Rating (MNR) of the Likert scale responses was done using the methods developed at McMaster University Canada by Johnson and Lavis [12]. Knowledge was classified as good at mean values between 3.00 and 5.00 while values less than 3.00 were categorized as poor. Attitude was classified as positive at mean values between 3.00 and 5.00 while values less than 3.00 were categorized as negative attitude [13-16]. Data entry and analysis was done using SPSS version 20. Descriptive statistics was used to summarize the data in relation to the different variables. Chi square statistics was used to determine the relationship between variables at 5% level of significance. Multivariable logistic regression analysis for predictors of knowledge and attitude towards cervical cancer screening was carried out. The cut-off point for including variables into the regression model was p<0.2 (20% level of significance). The adjusted odds ratio, level of significance and confidence interval for the independent variables were recorded and interpreted. Recordings from focus group discussions were transcribed verbatim and thematic analysis of relevant opinions were analysed manually. Some responses were quoted verbatim to capture the original ideas of the participants and informants.

**Ethical considerations:** ethical clearance for this study was obtained from the Research and Ethics Committee of Ebonyi State University, Nigeria with ref No: EBSU/DRIC/UREC/Vol.03/24. Only those respondents who gave their informed consent by signing the informed consent form participated in the study. The questionnaire did not include self-identifying characteristics and participation was voluntary. Written informed consent and permission to audio-record the FGDs was obtained from the participants before commencing the FGD.

## Results

**Knowledge of cervical cancer:** mean knowledge of cervical cancer among the respondents was 2.06±0.55. Only 0.4% and 2.9% of the respondents had very adequate and adequate knowledge on risk factors for cervical cancer respectively. Most respondents had very inadequate knowledge on the clinical features of cervical cancer (58.0%) and the screening tests available for its early detection (58.8%). A greater proportion of the respondents (58.6%) both strongly agreed and agreed that screening was useful in preventing cervical cancer (Table 1). The perspectives from FGD sessions also showed that the respondents had poor knowledge on cervical cancer. Majority of the participants admitted to having only limited knowledge but were aware of other cancers. One of the participants stated thus: *“I have heard of cancers generally but not that of the cervix, I haven´t heard of it or seen anyone affected except this one you are asking.”(FGD participant)*

**Table 1:** knowledge regarding cervical cancer and screening of married men in Nwezenyi

Knowledge item assessed	Very Inadequate/ Strongly disagree	Inadequate/Disagree	Fairly adequate/ Undecided	Adequate/Agree	Very Adequate/ Strongly Agree	Mean±SD	Median	Range
Knowledge of risk factors	139(56.7)	80(32.7)	18(7.3)	7(2.9)	1(0.4)	1.58±0.78	1.00	1-5
Knowledge of screening tests	144(58.8)	77(31.4)	16(6.5)	6(2.4)	2(0.8)	1.55±0.79	1.00	1-5
Knowledge of clinical features of cervical cancer	142(58.0)	81(33.1)	14(5.7)	7(2.7)	1(0.4)	1.55±0.77	1.00	1-5
Screening is useful in preventing cervical cancer	28(11.4)	28(11.4)	46(18.8)	62(25.3)	81(33.3)	3.57±1.35	4.00	1-5
Overall						2.06±0.55	2.00	1-5

None of them knew any of the risk factors or symptoms of cervical cancer. However one of the participants attempted to describe cancers as wounds but could not state any of the symptoms of cervical cancer. One of the men expressed concerns that his daughter´s longstanding illness could be due to this cancer. Their statements are below: *“Like one of us said, I don´t know how this cancer is contracted or how it affects people however this year, cancer killed a wife to one of our brothers residing in Awka. I was surprised to hear this and don´t even know how it is got.” (FGD participant). “To my understanding, cancer is like a wound or something like that, however I don´t know the symptoms of this cervical cancer.” (FGD participant). “This illness could be affecting one of my daughters who is sick, maybe she should be checked by you doctors to see if she has this disease.”* All the participants agreed that they needed to know more about cervical cancer and requested for more information from the researchers: *“We don´t know much about this cancer, you are just telling us now and we want to know more” (FGD participant)*.

The participants unanimously agreed that cervical cancer could be prevented but did not know how this could be achieved. One of the men admitted that their limited knowledge of the disease made him unsure of its preventability. Another expressed hopes that education/science should have a way of preventing it. They expressed their views thus: *“I don´t know if it can be prevented but I know of cancers of the breast and eye as I had a relative who had cancer of the eye and it was treated/prevented. It will be good for me if you explain more.”, “I think it can be prevented by those of you people who went to school.” (FGD participant)*.Although the men knew little about cervical cancer, there was general consensus that screening can prevent cervical cancer. One of the discussants stated it thus: *“I agree that screening can prevent this because with early detection the woman can be given the treatment and then she will be free.” (FGD participant)*.

**Attitude towards spousal support/involvement in cervical cancer screening:** the overall mean attitude towards male involvement in spouse´s cervical cancer screening was 3.86±0.51. Mean willingness to approve screening for spouse was 4.09±0.93 with majority (58.4%) willing to approve screening for their spouse respectively. Mean perception of importance of screening in cancer prevention was 4.04±0.81. Forty-five percent considered screening important for cervical cancer prevention, 31% considered it very important while 52 (21.2%) were undecided on its importance. Mean willingness to discuss screening with wife was 4.21±0.77. Sixty percent considered it important to discuss screening with their spouse while 33.9% considered it very important to do so. Mean perception on the importance of accompanying one´s spouse for screening was 3.86±1.08. Most respondents (51.0%) considered it important to accompany their spouse for screening while 26.9% considered it very important. Mean willingness to support spouse emotionally towards screening was 4.21±0.67. Most of the respondents (62.4%) considered it important to support their spouse emotionally towards screening. The mean willingness to pay for screening tests was 4.11±0.78 with majority of the men (64.1%) considering it important to pay for screening. The mean perceived spousal risk for cervical cancer was 2.49±1.17. Only 14.3% and 6.1% strongly agreed and agreed respectively that their spouse was at risk for cervical cancer (Table 2).

**Table 2:** attitude towards male involvement in cervical cancer screening among married men in Nwezenyi

Attitude items assessed^*^	GU/SU/SD/	UI/UW/D	UD/N	I/W/A	VI/SW/SA	Mean±SD	Median	Range
Willingness to approve screening for spouse	11(4.5)	7(2.9)	7(2.9)	143(58.4)	77(31.4)	4.09±0.93	4.00	1-5
Importance of screening in cancer prevention	2(0.8)	4(1.6)	52(21.2)	111(45.3)	76(31.0)	4.04±0.81	4.00	1-5
Willingness to discuss screening with spouse	4(1.6)	8(3.3)	4(1.6)	146(59.6)	83(33.9)	4.21±0.77	4.00	1-5
Importance of accompanying spouse for screening	15(6.1)	16(6.5)	23(9.4)	125(51.0)	66(26.9)	3.86±1.08	4.00	1-5
Willingness to support spouse emotionally towards screening	1(0.4)	7(2.9)	8(3.3)	153(62.4)	76(31.0)	4.21±0.67	4.00	1-5
Willingness to pay for screening tests	5(2.0)	8(3.3)	8(3.3)	157(64.1)	67(27.3)	4.11±0.78	4.00	1-5
My spouse is at risk of cervical cancer	55(22.4)	81(33.1)	59(24.1)	35(14.3)	15(6.1)	2.49±1.17	2.00	
Overall Attitude						3.86±0.51	3.57	

* GU- Grossly Unimportant, SD- Strongly Disagree, SU-Strongly Unwilling, UI-Unimportant, UW-Unwilling, D-Disagree, UD-Undecided, N-Neutral, I-Important, W-Willing, A-Agree, V1- Very Important, SW- Strongly Willing, SA-Strongly Agree

When the men who participated in the FGD were asked if husbands should support their wives towards cervical cancer screening, the positive attitude towards male involvement in cervical cancer screening seen from the quantitative aspect of the study was also reflected in their responses. All participants agreed that husbands should be involved in cervical cancer screening and that they would support screening for their spouses based on concerns that the woman may spread the disease to them or the children, would be unable to fund the screening by herself and fear of her death if cancer is left undiscovered. Their typical responses include the following: *“I will encourage her because I wouldn´t want the disease to spread to me or any of my children” (FGD participant). “It is good that husbands should be involved because who will pay for the test when it comes to money matters if not the husband?” (FGD participant). “I will discuss it thoroughly with her because if she dies, it will be big trouble. I will also accompany her to the health facility and go to any length for her wellbeing.” (FGD participant)*.

However, notwithstanding their positive attitude towards supporting their wives to screen for cervical cancer, all the discussants agreed that husbands would be angry and unsupportive of their wives if they were to do the screening test without obtaining their permission first even if it was following a health talk in the health facility. Reasons for insistence on wives obtaining spousal consent before screening were due to financial dependence of the women, male dominated decision-making and mutual obligation. One of the discussants emphasized the importance of inter-spousal discussion before screening. Their views were expressed thus: *“it is what I tell her that she will do so she shouldn´t do the (screening) test following a health talk without consulting me first. If I as her husband does not instruct her, then she should not try it.” (FGD participant). “Male involvement is very important as women listen to their husbands more than even the health care workers. They do whatever their husbands tell them as they see their husbands as their second”god´.” (FGD participant). “Since it is the man that will pay for the test, she should tell him first. Even if the wife has the money for the test, discussion with her husband before doing the test is important” (FGD participant). “Wives will also frown if their husband do such tests without their permission so she should get my permission first” (FGD participant)*.

The discussants were asked to state their views and reactions in the event that their wife had a positive cervical screening test result. Most of the participants were unwilling to accept that their wives could have a positive test result and hesitated in describing their reactions were this to occur. However (amidst giggles and laughter), all of them agreed that they would get themselves tested if their wives screening was suggestive of cervical cancer. Their responses are stated below: *“God forbid, it´s not my wife´s portion” (FGD participant). “Doctor, we are not praying for sickness so I don´t believe that my wife will have this kind of result.” (FGD participant). “I will go for my own test as well since I have sex with her.” (FGD participant)*.A greater proportion of respondents who had at least a senior secondary school education had good knowledge of cervical cancer screening compared to those who had at most a junior secondary school education (13.4% vs 5.6%; P=0.034). A higher proportion of respondents whose wives had ever screened for cervical cancer had good knowledge of cervical cancer screening compared to those whose wives had not been screened (50.0% vs 8.0%S; P=0.034) (Table 3). More respondents who were employed (97.6%) had a good attitude towards male involvement in cervical cancer screening compared to their unemployed counterparts (85.7%) and this difference in proportions was statistically significant (P=0.040) (Table 4). Men whose wives had ever been screened were 10.94 times more likely to have good knowledge towards cervical cancer screening compared to those whose wives had never been screened (OR = 10.94, 95% CI = 2.44-48.93; P=0.002) (Table 5).

**Table 3:** factors associated with knowledge on cervical cancer and screening among married men in Nwezenyi

Variable	Knowledge Frequency(%)		χ2	P--value
	Poor	Good		
**Age(years)**				
<40	111(88,8)	14(11.2)	0.985	0.321
≥40	111(92.5)	9(7.5)		
**Age of spouse(years)**				
<40	182(91.0)	18(9.0)	FT	0.584
≥40	40(88.9)	5(11.1)		
**Religion**				
Christianity	202(90.2)	22(9.8)	FT	0.703
Others**	20(95.2)	1(4.8)		
**Employment status**				
Unemployed	7(100.0)	0(0.0)	FT	1.000
Employed	215(90.3)	23(9.7)		
**Parity of spouse**				
≤5 children	121(89.6)	14(10.4)	0.341	0.559
>5 children	101(91.8)	9(8.2)		
**Educational status**				
Junior secondary and less	119(94.4)	7(5.6)	4.479	0.034*
Senior secondary and more	103(86.6)	16(13.4)		
**Family Type**				
Monogamy	175(91.1)	17(8.9)	FT	0.597
Polygamy	47(88.7)	6(11.3)		
**Wife ever screened for cervical cancer**				
Yes	4(50,0)	4(50.0)	FT	0.003*
No	218(92.0)	10(8.0)		

^*^ Statistically significant FT: Fishers Exact ^**^Traditional religion, Islam

**Table 4:** factors associated with attitude towards male involvement in cervical cancer screening among married men in Nwezenyi (n=245)

Variable	Attitude Frequency(%)		χ^2^	P-value
	Poor	Good		
**Age(years)**				
<40	2(1.6)	123(98.4)	FT	0.439
≥40	4(3.3)	116(96.7)		
**Age(years)**				
<40	5(2.5)	195(97.5)	FT	1.000
≥40	1(2.2)	44(97.8)		
**Religion**				
Christianity	5(2.2)	219(97.8)	FT	0.419
Others^	1(4.8)	20(95.2)		
**Employment status**				
Unemployed	1(14.3)	6(85.7)	FT	0.04^*^
Employed	5(2.1)	239(97.6)		
**Parity of spouse**				
5 or less children	3(2.2)	107(97.3)	FT	1.000
6 or more children	3(2.7)	239(97.6)		
**Educational status**				
≤Junior secondary school	4(3.2)	122(96.8)	FT	0.684
≥Senior secondary school	2(1.7)	117(98.3)		
**Family Type**				
Monogamy	4(2.1)	188(97.9)	FT	0.613
Polygamy	2(3.8)	51(96.2)		
**Wife ever screened for cervical cancer**				
Yes	0(0.0)	8(100.0)	FT	1.000
No	6(2.5)	231(97.5)		
**Knowledge on cervical cancer**				
Poor	6(2.7)	231(97.5)	FT	1.000
Good	0(0.0)	8(100.0)		

^*^ Statistically significant FT: Fishers Exact ^Others: Traditional religion, Islam

**Table 5:** logistic regression result for predictors of knowledge of cervical cancer and screening among married men in Nwezenyi

Independent variable	Adjusted Odds Ratio	95% CI for AOR		P value
		Lower	Upper	
**Educational status**				
≤Junior secondary school	1			
≥Senior secondary school	2.56	0.99	6.63	0.053
**Partner ever screened**				
No	1			
Yes	10.94	2.444	48.93	0.002^*^

* Statistically significant

## Discussion

This study assessed the knowledge and attitude towards cervical cancer screening among married men in a rural community in South-East Nigeria using a mixed methods study design. The study findings showed that the mean knowledge of cervical cancer and cervical cancer screening among the respondents was poor. Respondents exhibited inadequate knowledge of the risk factors, clinical features and screening tests for cervical cancer. This is consistent with findings from the focus group discussions as all the participants indicated that they had never heard of cervical cancer. Similar studies conducted among married and unmarried men have also found that the majority of the men surveyed were unaware of the causes, risk factors and screening tests for cervical cancer [8, 10, 17]. Likewise, limited knowledge on risk factors and clinical features of breast cancer has also been demonstrated in another study conducted among men [18]. These findings highlight the need to educate men on cervical cancer (and gynaecologic cancers generally) and its prevention given that they often act as key decision makers and financial providers for women to access health care in developing countries [19]. Respondents whose wives had ever screened for cervical cancer were likely to have good knowledge of the disease and its screening. This could be explained as their wives would have received some education on cervical cancer in the course of the screening and may have passed on some of these information to their husbands.

Majority of the respondents had a positive attitude towards male involvement in cervical cancer screening. This was evidenced by the degree of importance given to screening, the willingness to discuss, approve and sponsor cervical screening for their wives. This finding is in agreement with other similar studies conducted to assess the attitude of men towards involvement in cervical cancer screening [10, 11, 17, 20]. This favourable attitude of men towards involvement in reproductive health has been also documented in other studies on other reproductive health issues such as family planning and prevention of mother to child transmission of HIV [21-24]. To promote uptake of cervical cancer screening, it is important to leverage on this positive attitude of men to design and implement effective strategies that will involve them in the health of their wives and their families. Some reasons cited for supporting spousal cervical cancer screening by the FGD participants were concerns that the woman may spread the disease to them or the children, would be unable to fund the screening by herself and fear of wife´s death if cancer is left undiscovered.

In contrast, findings from another study conducted among both men and women revealed that some men considered screening for cervical unnecessary given the absence of symptoms and the costliness of screening tests [9]. This disposition could be because men tend to have poor health seeking behavior and prefer to only present to a doctor when they already have recognizable symptoms of a disease [25]. This unfavorable attitude towards screening because of the absence of symptoms could undermine their support for early detection of cervical cancer especially as screening should be done in apparently healthy symptom-free individuals. Interestingly, studies have shown that some women are also unwilling to screen for cervical cancer because they do not have symptoms [10]. Although the respondents in this study were willing to support screening for their wives, they emphasized during the FGDs that they would want their wives to obtain their approval first before accessing the screening services. Spousal disapproval has been cited as a barrier to the uptake of cervical cancer screening by African women [26]. This is mostly because of the cultural context of Africa (Nigeria inclusive) in which a married woman is obligated to obtain the consent of her husband before venturing into any activity or making any decision regarding her health. This perspective of men in such rural communities should be considered in programs that target uptake of cervical cancer screening by married women. Our findings also call for more research to understand the power dynamics in marital relationships and its influence on health seeking behavior and utilization of health services such as cervical cancer screening.

One of the outstanding findings of this study was that less than a third of the respondents perceived their spouse to be at risk of cervical cancer. This was corroborated by the FGD findings where most of participants vehemently rejecting the possibility of cervical cancer development in their spouses. This is particularly worrisome as some of their wives were grandmultiparous and lived in polygamous marital settings as evidenced by the socio-demographic characteristics. These factors have been identified as possible risk factors for cervical cancer [27]. The low risk perception by our respondents could be due to their low educational status and poor awareness on cervical cancer. Low risk perception by both men and women towards cervical cancer for their spouses and themselves respectively have been documented in other studies [11, 28]. More community-based awareness campaigns on cervical cancer with active participation of the menfolk should be carried out and these should highlight the risk factors associated with the cervical cancer. This will invariably improve uptake of cervical cancer screening services.

This study presents several strengths. Firstly, to the best of our knowledge, it is one of the few studies that have investigated the knowledge and attitude of men towards male involvement in cervical cancer screening and the only one to do so in Nigeria. Secondly, no respondent refused to complete the questionnaire, resulting in a high response rate. A main limitation of this study is the weakness of the self assessment technique used to assess the knowledge of cervical cancer. A more objective study design is recommended for future studies. This study was conducted in a rural community and this may act as a potential limitation to the generalization of the findings to married men in urban communities.

## Conclusion

This study has demonstrated that married men had limited knowledge of cervical cancer and poor perception of their wife´s risk for cervical cancer. In spite of their poor knowledge, the respondents demonstrated good attitude towards supporting their wives to be screened for cervical cancer. Their favorable disposition towards supporting spousal was however conditional on their wives first obtaining their consent before utilizing the screening services. There should be regular community-wide sensitization and awareness creation on cervical cancer prevention in order to improve their knowledge and approval for cervical cancer screening by their wives.

### What is known about this topic

- Cervical cancer morbidity and mortality is high in Nigeria;
- The uptake of cervical cancer screening is low in Nigeria;
- There is limited research evidence on male involvement in prevention of cervical cancer.

### What this study adds

- Knowledge of cervical cancer and screening is low among (married) men;
- Men are favourably disposed towards supporting their wives´ screening conditional on their pre-information and approval;
- This positive attitude towards cervical cancer screening can be harnessed in the design and implementation of programs to promote cervical cancer screening.
